# The Neurophysiological Basis of the Trial-Wise and Cumulative Ventriloquism Aftereffects

**DOI:** 10.1523/JNEUROSCI.2091-20.2020

**Published:** 2021-02-03

**Authors:** Hame Park, Christoph Kayser

**Affiliations:** ^1^Department for Cognitive Neuroscience, Faculty of Biology, Bielefeld University, D-33615 Bielefeld, Germany; ^2^Center for Cognitive Interaction Technology, Bielefeld University, D-33615 Bielefeld, Germany

**Keywords:** audiovisual, electroencephalography, multisensory, recalibration, spatial perception, ventriloquism aftereffect

## Abstract

Our senses often receive conflicting multisensory information, which our brain reconciles by adaptive recalibration. A classic example is the ventriloquism aftereffect, which emerges following both cumulative (long-term) and trial-wise exposure to spatially discrepant multisensory stimuli. Despite the importance of such adaptive mechanisms for interacting with environments that change over multiple timescales, it remains debated whether the ventriloquism aftereffects observed following trial-wise and cumulative exposure arise from the same neurophysiological substrate. We address this question by probing electroencephalography recordings from healthy humans (both sexes) for processes predictive of the aftereffect biases following the exposure to spatially offset audiovisual stimuli. Our results support the hypothesis that discrepant multisensory evidence shapes aftereffects on distinct timescales via common neurophysiological processes reflecting sensory inference and memory in parietal-occipital regions, while the cumulative exposure to consistent discrepancies additionally recruits prefrontal processes. During the subsequent unisensory trial, both trial-wise and cumulative exposure bias the encoding of the acoustic information, but do so distinctly. Our results posit a central role of parietal regions in shaping multisensory spatial recalibration, suggest that frontal regions consolidate the behavioral bias for persistent multisensory discrepancies, but also show that the trial-wise and cumulative exposure bias sound position encoding via distinct neurophysiological processes.

**SIGNIFICANCE STATEMENT** Our brain easily reconciles conflicting multisensory information, such as seeing an actress on screen while hearing her voice over headphones. These adaptive mechanisms exert a persistent influence on the perception of subsequent unisensory stimuli, known as the ventriloquism aftereffect. While this aftereffect emerges following trial-wise or cumulative exposure to multisensory discrepancies, it remained unclear whether both arise from a common neural substrate. We here rephrase this hypothesis using human electroencephalography recordings. Our data suggest that parietal regions involved in multisensory and spatial memory mediate the aftereffect following both trial-wise and cumulative adaptation, but also show that additional and distinct processes are involved in consolidating and implementing the aftereffect following prolonged exposure.

## Introduction

Sensory recalibration serves to continuously adapt perception to discrepancies in our environment, such as the apparent displacement of the sight and sound of an object (De Gelder and Bertelson, 2003; Chen and Vroomen, 2013). Despite the importance of such adaptive multisensory processes in everyday life, their neural underpinnings remain unclear. Our environment changes on multiple timescales, and, not surprisingly, perceptual recalibration also emerges on distinct scales (Bruns and Röder, 2015, 2019; Van der Burg et al., 2015; Bosen et al., 2017, 2018; Rohlf et al., 2020). During the ventriloquism aftereffect (Canon, 1970; Radeau and Bertelson, 1974; Recanzone, 1998; Wozny and Shams, 2011; Bruns and Röder, 2015), the exposure to displaced acoustic and visual stimuli in an audiovisual trial reliably biases the perceived location of subsequent sounds received during a unisensory trial (Woods and Recanzone, 2004; Frissen et al., 2012; Mendonça et al., 2015; Watson et al., 2019). This aftereffect increases with cumulative exposure to a consistent discrepancy, but seems to independently emerge trial by trial and following prolonged exposure (Frissen et al., 2012; Bruns and Röder, 2015; Van der Burg et al., 2015; Watson et al., 2019; Kramer et al., 2020). The trial-wise and cumulative biases differ in their specificity to the sensory features of the inducing stimuli and were suggested to arise from distinct neurophysiological correlates (Bruns and Röder, 2015, 2019). Still, this hypothesis has not been directly tested.

In a previous study on the ventriloquism aftereffect, we showed that medial parietal regions integrate audiovisual information within a trial and mediate the trial-by-trial aftereffect (Park and Kayser, 2019), implying a role of parietal regions involved in spatial working memory and multisensory causal inference in trial-wise recalibration. Given that cumulative recalibration results from the prolonged exposure to consistent sensory discrepancies, and the cumulative effect hence encompasses trial-wise effects at least to some degree, one could reason that the same parietal regions also mediate a cumulative effect. The few existing neuroimaging studies reported correlates in early sound-evoked potentials and near early auditory cortices (Bruns et al., 2011; Zierul et al., 2017), and concluded that the cumulative aftereffect is implemented by early sensory regions, in line with evidence from single-cell recordings (Recanzone, 1998; Recanzone et al., 2000). However, these studies focused on neural signatures of sound encoding during the unisensory test trial to probe for neural correlates, thus possibly biasing the findings toward auditory pathways. Indeed, one study also reported changes in the functional coupling between auditory and parietal regions, hinting at a more extensive cerebral network shaping the cumulative effect (Zierul et al., 2017).

We set out to directly compare the neurophysiological correlates of audiovisual spatial recalibration on a trial-by-trial level [short-term (ST)] and after cumulative exposure [long-term (LT)] in human participants, using the same stimuli and design for both paradigms. Following our previous work (Park and Kayser, 2019), we combined a ventriloquism task with temporally precise neuroimaging [electroencephalography (EEG)] and applied single-trial neurobehavioral modeling. We focused on the hypothesis that the trial-wise and cumulative aftereffects arise from a partly shared substrate (in particular, medial parietal regions) and implemented two analysis strands to test this. One strand capitalized on the cerebral representations that reflect the discrepant multisensory evidence received during the audiovisual trial, hence directly focused on the multisensory processes that guide the adaptive behavior (Canon, 1970; Radeau and Bertelson, 1974, 1977; Wozny and Shams, 2011). Another strand characterized the cerebral representations reflecting the task-relevant acoustic information in the auditory trial and asked whether and when these representations are biased by the previously experienced multisensory discrepancy. This analysis follows the logic set out previously (Bruns et al., 2011) and directly investigates how previous multisensory exposure shapes the aftereffect in the trial where it manifests in behavior (Zierul et al., 2017; Park and Kayser, 2019).

## Materials and Methods

### 

#### 

##### Participants

Twenty right-handed healthy young adults (age range, 22–39 years; mean ± SD age, 26.7 ± 4.20 years; 10 females) participated in the study. The sample size was planned based on generic recommendations for behavioral studies (Simmons et al., 2011) and matched that used in similar magnetoencephalography (MEG)/EEG studies on the ventriloquism aftereffect (Bruns et al., 2011; Park and Kayser, 2019). All participants reported normal vision and hearing, with no history of neurological or psychiatric disorders, and each provided written informed consent and was compensated for their time. The study was approved by the local ethics committee of Bielefeld University. One participant's data were excluded because of not being able to follow the task instructions. Therefore, we report data from 19 participants.

##### Stimuli

The acoustic stimulus was a 1300 Hz sine wave tone (50 ms duration) sampled at 48 kHz and presented at 64 dB root mean square through one of five speakers (MKS-26/SW, MONACOR International), which were located at five horizontal locations [−23.2°, −11.6°, 0°, 11.6°, 23.2° (vertical midline = 0°; negative = left; positive = right)]. Sound presentation was controlled via a multichannel soundcard (Creative Sound Blaster Z) and amplified via an audio amplifier (t.amp E4-130, Thomann). Visual stimuli were projected (Predator Z650, Acer) onto an acoustically transparent screen (2 m × 1 m; Modigliani, Screen International), which was located at 135 cm in front of the participant. The visual stimulus was a cloud of white dots distributed according to a two-dimensional Gaussian distribution (*N* = 200 dots; SD of vertical and horizontal spread, 2°; width of a single dot, 0.12°; duration, 50 ms). Stimulus presentation was controlled using the Psychophysics toolbox (Brainard, 1997) for MATLAB (MathWorks) with ensured temporal synchronization of auditory and visual stimuli.

##### Paradigm and task

**Figure 1.**
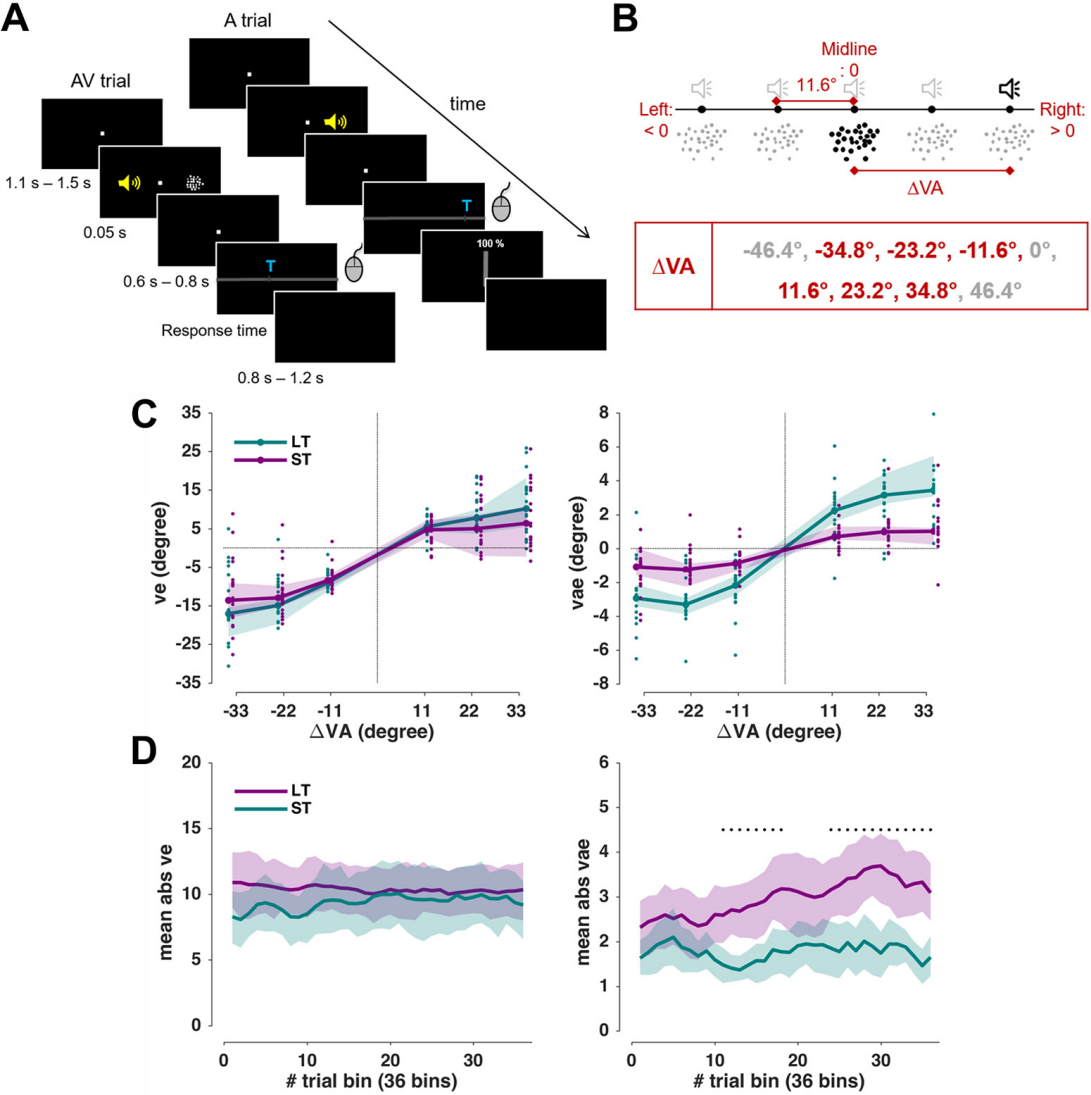
Experiment setup and behavioral data. ***A***, Example sequence of AV and A trials (rare V trials are not shown). The yellow speaker is for illustration only; the sound came from speakers placed behind the screen. The participants submitted their response by moving a mouse cursor to the location where they perceived the sound. The confidence rating was only taken in the A trial. ***B***, ΔVA is the distance between the visual and sound stimuli, each located at one of five horizontal locations. Among the nine possible values of ΔVA, only six were used for efficiency. ***C***, Behavioral results: the ventriloquism effect (left) and ventriloquism aftereffect (right), both median values across participants (*n* = 19); the shaded areas are 95% confidence intervals around the median. Dots show individual participant's data. ***D***, Temporal progression of biases. Shaded areas indicate 95% hybrid bootstrap confidence intervals around the mean. Black dots denote a significant difference between the LT and ST tested with a cluster-based permutation test (*p* < 0.01; for details, see Materials and Methods). ve: trial-wise ventriloquism effect. vae: trial-wise ventriloquism aftereffect. LT: long-term paradigm. ST: short-term paradigm.

The paradigm was based on a single-trial audiovisual localization task (Wozny and Shams, 2011; Park and Kayser, 2019), with trials and conditions designed to probe both the ventriloquism effect and the ventriloquism aftereffect. Participants were seated in front of an acoustically transparent screen with their heads on a chin rest. They were instructed not to move their head while performing the task. Five speakers were located immediately behind the screen, and participants responded with a mouse. Their task was to localize a sound during either audiovisual (AV; sound and visual stimulus presented simultaneously) or auditory (A: only sound) trials, or to localize a visual stimulus during visual trials (V, only visual stimulus). For the AV trials, the locations of auditory and visual stimuli were drawn semi-independently from the five locations to yield six different audiovisual discrepancies (ΔVA; −34.8°, −23.2°, −11.6°, 11.6°, 23.2°, and 34.8°). Of nine possible ΔVAs, we excluded the far left/right extremes and the colocated condition (0°) to economize time of session (Fig. 1*B*). For A or V trials, stimulus locations were drawn from five locations randomly.

##### Experimental setup

Each participant underwent two sessions on different days in pseudorandomized order, as follows: one for LT and one for ST recalibration. The LT paradigm comprised two parts, three consecutive leftward recalibration blocks, in which the audiovisual discrepancy was always negative (ΔVA: <0°: −34.8°, −23.2°, −11.6°), and three consecutive rightward recalibration blocks in which the discrepancy was always positive (ΔVA: >0°: 11.6°, 23.2°, 34.8°). The leftward and rightward blocks were separated by a brief break. Other than the negative/positive constraint, the positions of the acoustic and visual stimuli were chosen randomly. The ST paradigm comprised five blocks, with each block featuring all six audiovisual discrepancies in random sequence. Each ΔVA was repeated 72/60 times for LT/ST, respectively. There were 432 AV trials, 432 A trials, and 72 V trials for the LT. There were 360 AV trials, 360 A trials, and 55 V trials for the ST. The A trial always came immediately after the AV trial. The V trials were interleaved to maintain attention (V trials always came after A trials, thus not interrupting the AV–A sequence). The LT paradigm included more trials than the ST paradigm to account for the “buildup” of the recalibration bias. However, we entered all trials in the data analysis, and verified that the main results would not change when excluding the first 12 trials from each of the LT blocks. The order of trials was pseudorandomized with the constraint that the AV trial is always followed by the A trial, and the (few) V trial always comes after the A trial. Each trial started with a fixation period (uniform, 1100 ms–1500 ms), followed by the stimulus (50 ms). After a random post-stimulus period (uniform, 600 ms–800 ms), a horizontal bar was shown, along which participants could move a cursor (Fig. 1*A*). A letter indicated which stimulus participants had to localize. On the A trials, participants also reported their confidence by moving a vertical bar between 0% and 100%. We did not analyze the confidence for this study as these were outside the scope of the specific hypotheses addressed here. There were no constraints on response times; however, participants were instructed to respond intuitively, and to not dwell on their response. Intertrial intervals varied randomly (uniform, 800 ms–1200 ms). Participants were asked to maintain fixation during the entire trial except the response, during which they could freely move their eyes. Eye-tracking data were acquired with a head-mounted eyetracker (EyeLink II, SR Research) at a frequency of 200 Hz. Saccadic eye movements were detected using the “cognitive” setting in the EyeLink II software.

##### Analyses of behavioral data

The trial-wise ventriloquism effect (ve) in the AV trials was defined as the difference between the actual sound location (A_AV_) and the reported location (R_AV_): ve = R_AV_ − A_AV_. The trial-wise ventriloquism aftereffect (vae) in the A trials was defined as the difference between the reported location (R_A_) and the mean reported location for all A trials of the same stimulus position (μR_A_; i.e., vae = R_A_ − μR_A_). This ensured that any intrinsic general bias in sound localization would not influence this measure (Wozny and Shams, 2011).

To quantify the dependency of both biases (ve, vae) on the ΔVA, we used general linear modeling. In particular, we included a linear and a nonlinear dependency (square root of ΔVA) of the bias on ΔVA and asked whether the respective slopes differ between the LT and ST paradigms. The nonlinear dependency was included following predictions from multisensory causal inference models, which posit that the perceptual bias decreases when the stimuli are sufficiently far apart and do not seem to originate from a common source (Körding et al., 2007; Rohe and Noppeney, 2015; Cao et al., 2019). We fit a generalized linear mixed-effects model across all trials from all participants and paradigms. This model included the paradigm and its interaction with the discrepancy terms and by including participants (subj) as random effects to directly compare the group-level biases between LT and ST paradigms, as follows:

(1) $$\text{Bias }∼\beta_{0}+\beta_{\text{1}}\cdot {\left(\Delta \text{VA}\right)}^{½}+\beta_{\text{2}}\cdot \Delta \text{VA }+\beta_{\text{3}}\cdot \text{P }+\beta_{\text{4}}\cdot {\left(\Delta \text{VA}\right)}^{½}:\text{P}+\beta_{5}\cdot \Delta V\text{A}:\text{P}+\left(1/\text{subj}\right),$$

where Bias can be ve or vae, P is the paradigm (LT or ST, coded as categories). Note that here and in the following, (ΔVA)^½^ stands for the signed square root of the magnitude of ΔVA (i.e., sign(ΔVA) * sqrt(abs(ΔVA)).The coefficients β_1_, β_2_ quantify the group-level biases, and β_4_, β_5_ quantify their interactions with the paradigm. Fitting was performed using a maximum likelihood procedure in MATLAB R2017a (fitglme.m).

As previous work has shown that the preceding response can potentially be a driving factor for the ventriloquism aftereffect (Park et al., 2020), and because serial dependencies in perceptual choices prevail in many laboratory paradigms (Fritsche et al., 2017; Kiyonaga et al., 2017; Talluri et al., 2018), we tested whether including the previous response (i.e., R_AV_) would improve the predictive power of model 1 (Eq. 1), as follows:

(2) $$\text{vae }∼\beta_{0}+\beta_{\text{1}}\cdot {\left(\Delta \text{VA}\right)}^{½}+\beta_{\text{2}}\cdot \Delta \text{VA }+\beta_{\text{3}}\cdot {\text{R}}_{\text{AV}}+\beta_{\text{4}}\cdot \text{P}+\beta_{\text{5}}\cdot {\left(\Delta \text{VA}\right)}^{½}:\text{P}+\beta_{6}\cdot \Delta \text{VA}:\text{P}+\left(1/\text{subj}\right).$$

We compared the two models (Eqs. 1, 2) based on their respective Bayesian information criteria (BICs). We also inspected the temporal progress of the perceptual biases (ve, vae) and compared these between the two paradigms (Fig. 1*D*). The LT data were averaged in increments of five trials, resulting in 36 bins, and the binned data were combined across blocks with leftward and rightward adaptation. ST data were averaged in increments of nine trials, resulting in 36 bins.

##### EEG acquisition and preprocessing

EEG data were recorded using an active 128-channel system (BioSemi), with an additional 4 electrodes placed near the outer canthi and below the eyes to record the electro-oculogram (EOG). Electrode offset was <25 mV. Offline preprocessing and analyses were performed with MATLAB R2017a (MathWorks) using the Fieldtrip toolbox (version 20190905; Oostenveld et al., 2011). The data were bandpass filtered between 0.6 Hz and 90 Hz, resampled to 150 Hz and epoched from −0.8 s to 0.65 s around stimulus onset (0 s). Noise removal was performed using independent component analysis (ICA) simultaneously across all blocks recorded on the same day. The ICA was computed based on 40 principal component analysis components. We removed ICA components that reflect eye movement artifacts, localized muscle activity or poor electrode contacts (mean ± SD;17.2 ± 4.45 rejected components per participant). These were identified as in our previous studies (Kayser et al., 2017; Grabot and Kayser, 2020) following definitions provided in the literature (O'Beirne and Patuzzi, 1999; Hipp and Siegel, 2013). Additionally, trials with amplitude exceeding 175 mV were rejected. 0.9% ± 2.6% (mean ± SD) of the total trials was rejected across all participants.

##### EEG discriminant analysis

**Figure 2.**
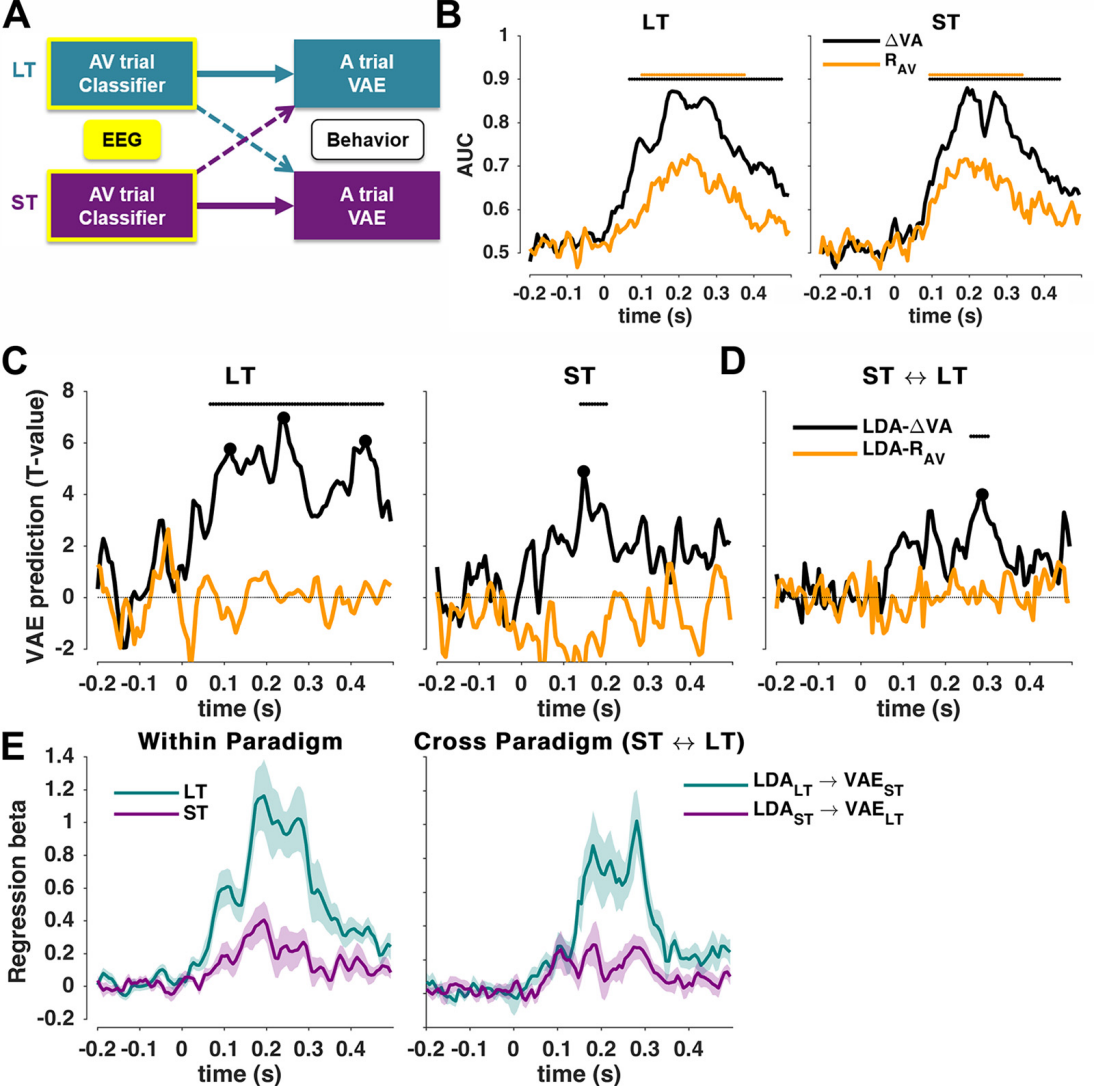
Predicting the trial-wise aftereffects based on neurophysiological representations. ***A***, In two separate analyses, we quantified the predictive power of EEG-derived representations of either the multisensory discrepancy or the response in the AV trial to predict the trial-wise vae bias in the A trial, either (1) within a paradigm (thick arrows) or (2) across paradigms (dotted arrows). ***B***, Classifier performance (group-level mean, *n* = 19) for both paradigms (ST and LT) as cross-validated area under the ROC curve (AUC). ***C***, Neurobehavioral models predicting the trial-wise aftereffect within paradigms based on the EEG-derived cerebral encoding of sensory (ΔVA) or motor (R_AV_) variables in the AV trial. Graphs show group-level *t*-maps of the underlying regression betas. ***D***, Neurobehavioral models predicting the aftereffect across paradigms. Horizontal solid lines denote significance based on cluster-based permutation-based statistics (*p* < 0.01; see Materials and Methods). ***E***, Time course of regression betas (for LDA_ΔVA) for the same data as in ***C***. Left, Within-paradigm analyses. Right, Cross-paradigm analyses. Solid lines indicate the group-level mean, shaded areas are SEM across participants.

**Figure 3.**
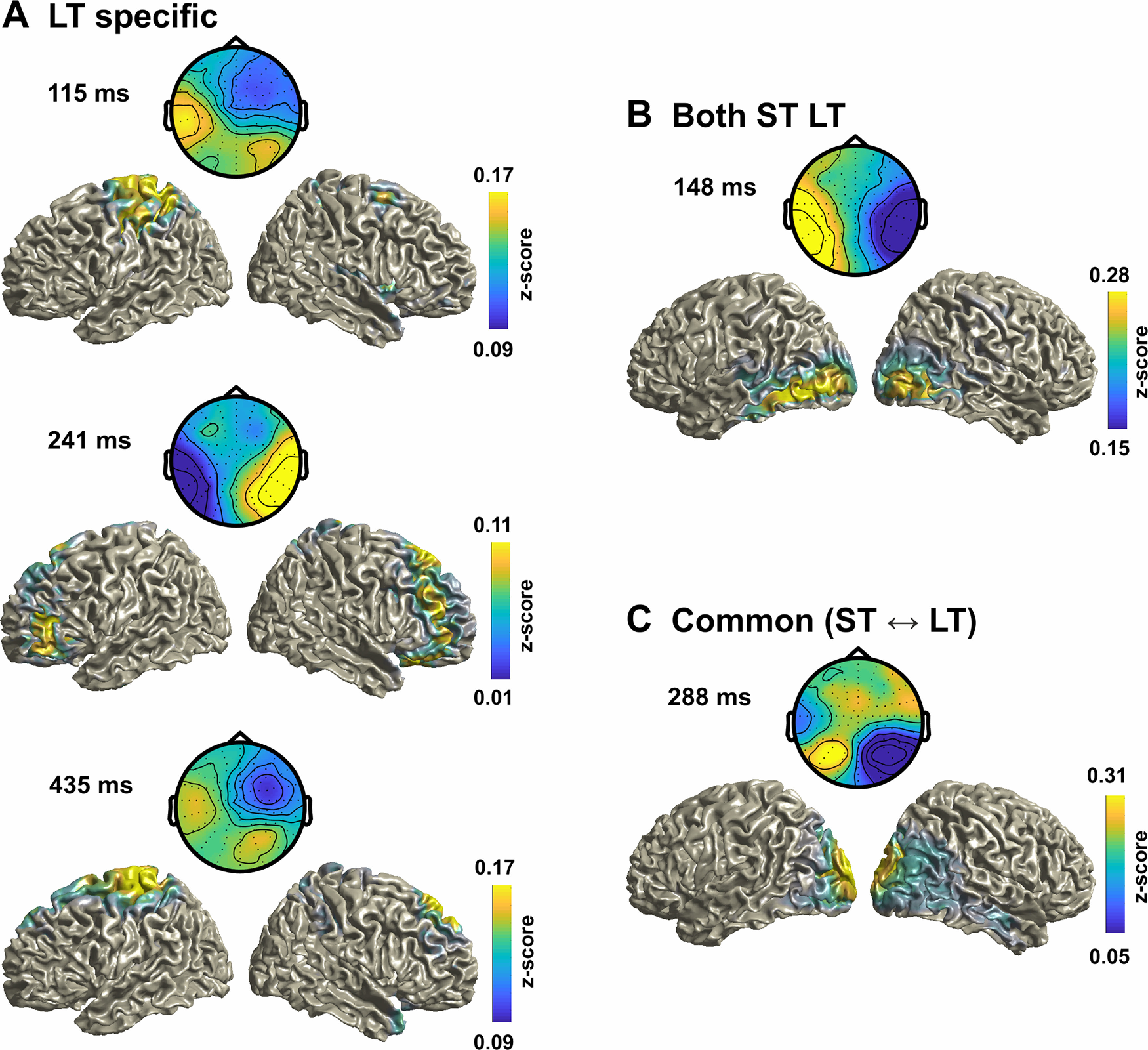
EEG topographies and source maps for the LDA-ΔVA classifier. ***A***, Group-averaged topographies (forward models) and source maps for the three LT-specific time points derived in Figure 2*C*, left. ***B***, ***C***, Time point common to both paradigms (Fig. 2*C*, right; ***B***), and for the peak time point in the cross-paradigm analysis in Figure 2*D* (***C***). The data are shown as *z* score-transformed correlations between single trial source activity and the LDA output (see Materials and Methods). For ***B*** and ***C***, the correlations were averaged across paradigms.

**Figure 4.**
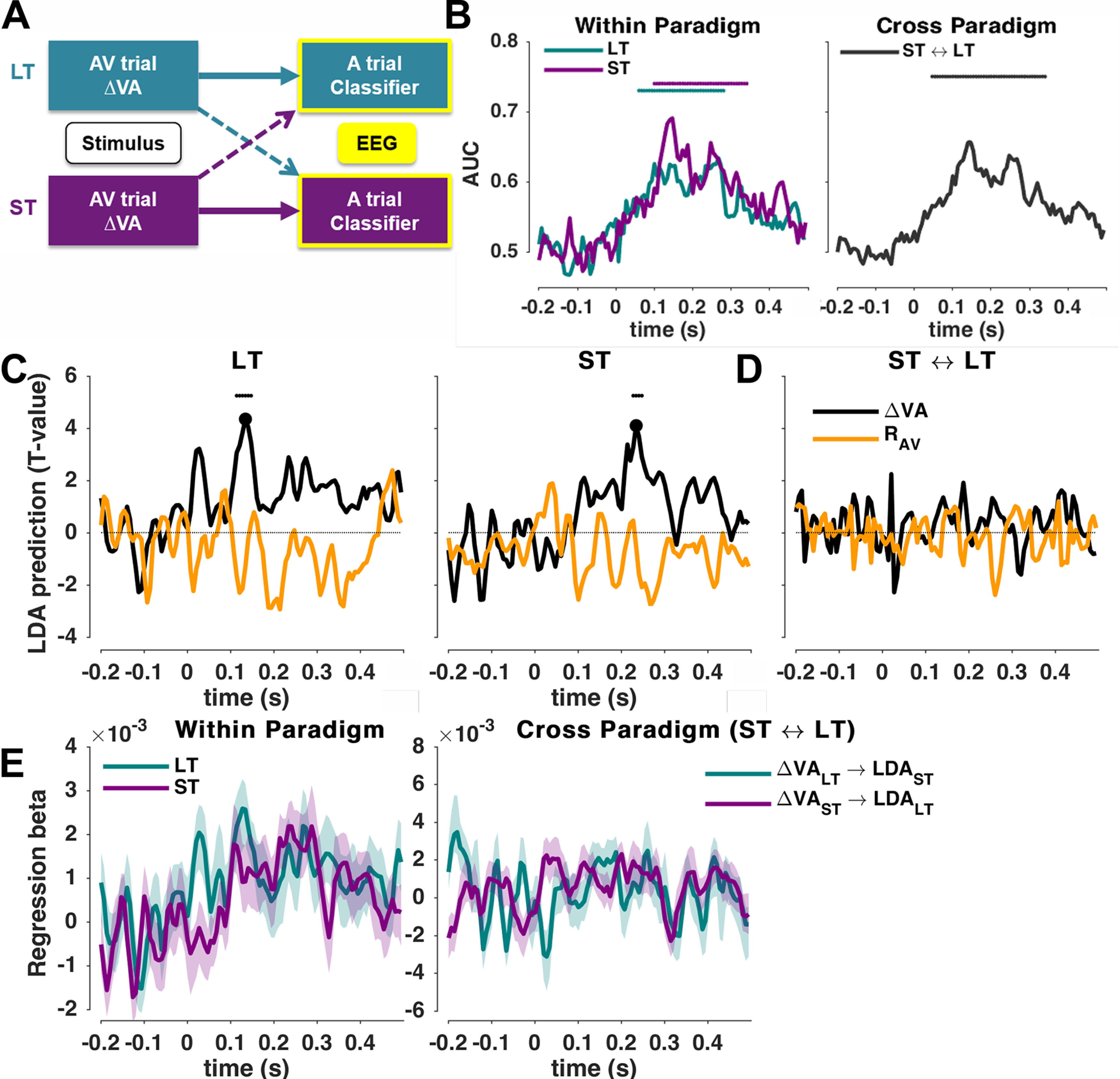
Predicting the neurophysiological representations in the A trial based on the previous stimuli. ***A***, In two separate analyses, we quantified the predictive power of the multisensory discrepancy (or motor response) in the AV trial to predict the trial-wise neurophysiological representations of sound location in the A trial, either (1) within a paradigm (thick arrows) or (2) across paradigms (dotted arrows). ***B***, Classifier performance for sound location (group-level mean, *n* = 19) for (left) both paradigms (ST and LT) or across paradigms (right) as cross-validated area under the ROC curve (AUC). ***C***, Models reflecting the influence of the multisensory discrepancy (ΔVA) or motor (R_AV_) variables in the AV trial on the trial-wise representations of sound location, within each paradigm. Graphs show group-level *t*-maps of the underlying regression betas. Significance based on cluster-based permutation-based statistics (*p* < 0.05; see Materials and Methods). ***D***, Same analysis computed across paradigms. ***E***, Time course of regression betas for the results for ΔVA in ***C*** and ***D***. Solid lines indicate the group-level mean; shaded areas are SEM values across participants.

**Figure 5.**
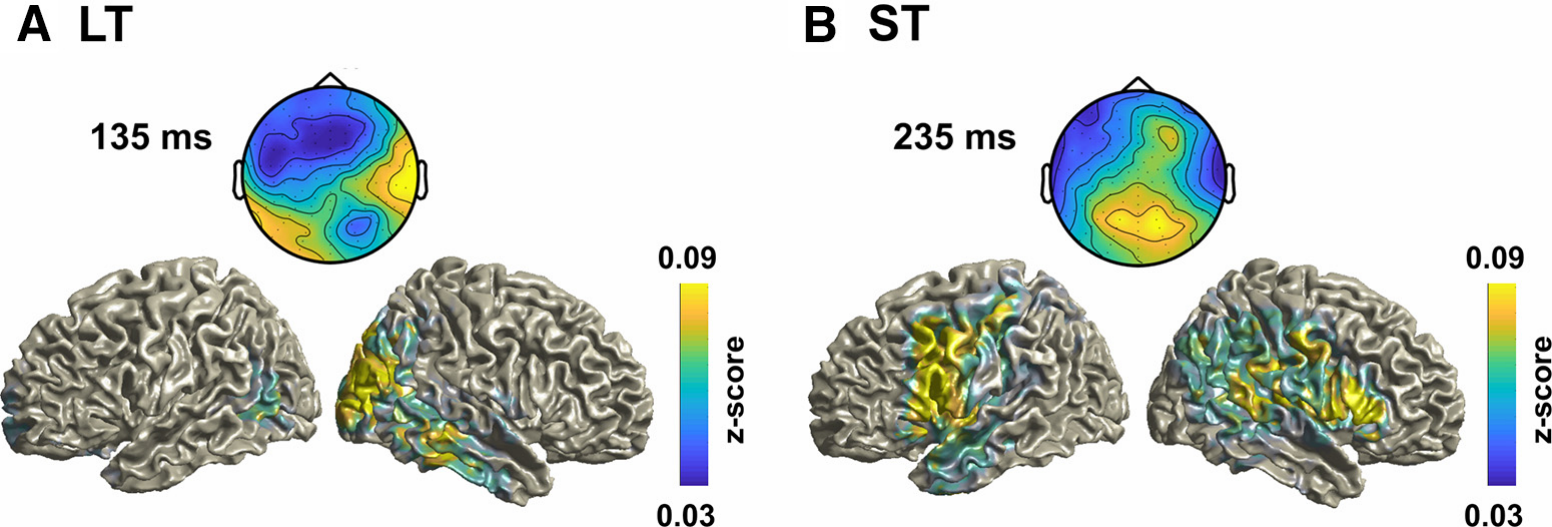
EEG topographies and source maps for the LDA-A_A_ classifier. ***A***, ***B***, Group-averaged topographies (forward models) and source maps for the LT-specific time point (Fig. 4*C*, left; ***A***), and for the ST-specific time point (Fig. 4*C*, right; ***B***). The data are shown as *z* score-transformed correlations between single-trial source activity and the LDA output (see Materials and Methods). Source maps were averaged across both paradigms given that the LDA forward models were significantly correlated between paradigms.

To extract neural signatures of the encoding of different variables of interest, we applied a cross-validated regularized linear discriminant analysis (LDA; Parra and Sajda, 2003; Blankertz et al., 2011) to the single-trial data from the AV trials (Figs. 2, 3) or the A trials (Figs. 4, 5). Preprocessed EEG data were filtered between 2 Hz and 40 Hz (fourth-order Butterworth filter), and the LDA was applied to the data aligned to stimulus onset (0 s) in 40 ms sliding windows, with 6.7 ms time steps (time window, approximately −0.4 s to 0.5 s). The regularization parameter was set to 0.1, as in previous work (Park and Kayser, 2019).

We computed separate linear discriminant classifiers for the different variables of interest: (1) the multisensory discrepancy in the AV trial (ΔVA); (2) the response in the AV trial (R_AV_); and (3) the sound location in the A trial (A_A_). For each variable, we classified whether that variable was left or right lateralized by grouping the single trial values into left (<0°) or right (>0°), similar to our previous study (Park and Kayser, 2019). Importantly, by binarizing the variables, we avoided specific assumptions about whether the aftereffect follows a linear or nonlinear dependency on the AV discrepancy. The classifier performance (Figs. 2*B*, 4*B*) was characterized as the area under the curve (AUC) of the receiver operating characteristic (ROC) obtained from sixfold cross-validation, training the classifier on five-sixths of the data and computing the AUC on the remaining one-sixth. We derived scalp topographies for each classifier by estimating the corresponding forward model, defined as the normalized correlation between the discriminant component and the EEG activity (Parra et al., 2005).

##### Neurobehavioral models predicting the trial-wise aftereffect

We then used these classifiers to elucidate the correlates of the single-trial vae biases. We implemented two strands of analysis that differed in their overall goals. In a first strand, we focused on brain activity during the AV trial and used classification to extract cerebral representations of the multisensory discrepancy (ΔVA), or the response in that trial (R_AV_). We then asked which cerebral representations of the audiovisual disparity (or response) are predictive of the response bias in the subsequent unisensory trial. We first tested this within each paradigm separately and then probed whether the relevant representations of disparity (response) are possibly the same between paradigms. For this, we implemented the following two analyses that differed in the trials used to train the classifier and the trials used to predict the behavioral bias: first, we tested the ability to predict the vae bias, obtained in the A trial, based on the EEG activity obtained in the preceding AV trial within each paradigm (Fig. 2*A*, thick arrows); second, we tested the ability to cross-predict the vae bias in one paradigm (e.g., ST) based on the brain activity in the AV trials of the other paradigm (LT; Fig. 2*A*, dotted arrows). This cross-classification analysis directly tests the assumption that the cerebral activations (here captured by the classifier weights) representing the audiovisual discrepancy and driving the aftereffect are identical across paradigms both in their spatial generators and in time relative to the presentation of the AV stimulus.

We computed two linear models for each of the two analyses (within or between paradigms), with LDA-ΔVA (or LDA-R_AV_) standing for the respective continuous single-trial classifier predictions, which provides a proxy to the cerebral representation of the respective variable of interest (Parra et al., 2005; Philiastides et al., 2014; Kayser et al., 2016; Park and Kayser, 2019; Kayser and Kayser, 2020):

(3) $${\text{vae}}_{P}∼\beta_{0}+\beta_{\text{1}}\cdot {\text{LDA}}_{\text{P}}\text{-}\Delta \text{VA}$$

(4) $${\text{vae}}_{P}∼\beta_{0}+\beta_{\text{1}}\cdot {\text{LDA}}_{\text{P}}\text{-}{\text{R}}_{\text{AV}},$$

where P denotes paradigms (LT, ST). From the coefficients (β*_1_*) obtained for individual participants we then determined (1) whether the cerebral encoding of a variable offered significant predictive information for the vae by testing the coefficient at the group-level against zero, (2) when this prediction emerged, and (3) by looking at the forward models of the respective LDAs, we determined the underlying cerebral sources.

These models were computed using EEG activity in the AV trial based on threefold cross-validation. That is, we trained the LDA classifier on one training fold of the data (e.g., in the LT paradigm), used the respective weights to predict the classifier output in the testing fold (either in the LT data for a within-paradigm analysis, or in the ST data for cross-classification), and then computed the regression models (Eqs. 3, 4) between the predicted classifier activity and the vae bias on this testing fold. We averaged the resulting beta values across 30 repeats of this analysis. The use of cross-validation is only necessary for the within-paradigm analysis. However, to keep the two analyses comparable, we used the same approach for both the within-paradigm and between-paradigm analysis. A threefold cross-validation was used (rather than a higher number of folds; e.g., as used to compute the AUC) to enter more trials in the neurobehavioral regression, which yielded more robust results. Finally, we derived group-level *t* values for the coefficients for each predictor at each time point, and assessed their significance using cluster-based permutation statistics controlling for multiple comparisons (see Statistical analysis, below).

In a second analysis strand, we focused on the brain activity during the A trial and used classification to extract cerebral representations of the sound location presented in that trial (Fig. 4). We then asked where these representations of the task-relevant acoustic information are shaped by the previously experienced discrepancy or the response (R_AV_). To this end we computed the following regression model:

(5) $$L\text{D}A_{\text{P}}\text{-}{\text{A}}_{A}∼{\text{β}}_{0}+{\text{β}}_{1}\cdot \text{Δ}V\text{A}$$

(6) $$\text{L}D{\text{A}}_{P}\text{-}{\text{A}}_{A}∼{\text{β}}_{0}+{\text{β}}_{1}\cdot {\text{R}}_{\text{AV}}.$$

Similar to the first analysis strand, we derived these models using threefold cross-validation, establishing the classifier weights on a testing fold and deriving classifier predictions and the regression model on the testing fold, averaging the resulting coefficients over 30 cross-validation sets of trials. Again, we implemented this analysis once within each paradigm separately, and once cross-testing between paradigms, analogously to the first analysis strand (Fig. 4*B*).

##### EEG source analysis

Single-trial source signals were derived using a linear constrained minimum variance beamformer (7% normalization, using a covariance matrix obtained from −0.6 s to 0.5 s peristimulus period, projecting along the dominant dipole orientation) as implemented in the FieldTrip toolbox (Oostenveld et al., 2011). As participant-specific anatomic data were not available, we used a standardized head model using the average template brain of the Montreal Neurologic Institute. Lead fields were computed using a grid spacing of 6 mm. Then, we computed the source-level correlation between the single-trial grid-wise source activity for each participant and the LDA output activity over trials to quantify the relevant source regions at specific time points, similar to obtaining the forward scalp distributions by correlating the sensor and LDA components (Parra et al., 2005; Haufe et al., 2014). Source correlations were *z*-scored before averaging across participants. To interpret these group-level source maps, we thresholded these above the 95th percentile, and identified clusters with a minimum cluster size of 80 voxels based on a connected components algorithm (SPM8 toolbox, 2008, Wellcome Trust Center for Neuroimaging). We then extracted the anatomic labels based on the Automated Anatomical Labeling (AAL) atlas (Tzourio-Mazoyer et al., 2002), to determine those regions covered by these clusters (reporting atlas regions containing at least 20 voxels and occupying at least 30% of the total number of voxels for each atlas region, excluding deep structures such as the thalamus or cerebellum).

##### Eye movement analyses

We performed three analyses to rule out potential confounds arising from systematic eye movements. First, we computed the number of saccades between −50 ms and 100 ms around stimulus onset that were >1° visual angle in the A trials. Then, we computed the percentage of saccades in AV trials between the stimulus onset and +400 ms that pointed in the same direction as ΔVA. Finally, to rule out the possibility that eye movements contribute by inducing specific artifacts in the EEG signals, we applied the neurobehavioral analyses to the EOG data rather than the EEG data.

##### Statistical analysis

To test the (trial-averaged) ve and vae from zero, we used a signed-rank test, correcting for multiple tests using the Holm procedure with a familywise error rate of *p* < 0.05 (Fig. 1*C*). The confidence intervals (CI) for the median/mean (Fig. 1*C*,*D*) were obtained using the bootstrap hybrid method with 199 resamples (Bootstrap MATLAB Toolbox; Zoubir and Boashash, 1998). Group-level inference on the LDA time course was performed using randomization procedures and cluster-based statistical enhancement controlling for multiple comparisons along time (Nichols and Holmes, 2002; Maris and Oostenveld, 2007). First, we shuffled the sign of the true single-participant effects (the signs of the chance-level corrected AUC values; or the signs of single-participant regression beta values) and obtained distributions of group-level effects (mean for AUC, *t* values for regression models) based on 3000 randomizations. We then applied spatial clustering based on a minimal cluster size of four and using the sum as cluster statistics. For testing the LDA performance, we thresholded the first-level effects based on the 99th percentile (i.e., *p* < 0.01) of the full distribution of randomized AUC values. For testing regression betas, we used parametric thresholds corresponding to a two-sided *p* < 0.01 (tcrit (critical t-value) = 2.81, df = 18). The threshold for determining significant clusters was *p* < 0.01 (two sided), although we also inspected a more lenient threshold of *p* < 0.05. We tested for significant temporal clusters for classifier performance in the whole time window of interest (−0.4 s to 0.5 s), while the neurobehavioral models were restricted to a time window of interest within the significant discriminant performance for the respective variable (ΔVA, R_AV_). For the cross-paradigm analyses, we computed the conjunction statistic obtained at each time point by taking the smaller of the two *t* values obtained from LDA_ST_ → vae_LT_ and LDA_LT_ → vae_ST_ (Eqs. 3, 4; Nichols et al., 2005).

To compare the similarities of the group-level forward models of the LDA classifiers obtained in different paradigms, or at different time points, we quantified their group-level similarity using Pearson correlation. Statistical significance was tested using bootstrapping over the (random) selection of participants used to compute the group-level mean (at *p* < 0.01, using 3000 resamples).

## Results

### Behavioral biases

Behavioral responses in AV trials revealed a clear ventriloquism bias as a function of the audiovisual discrepancy (ΔVA = V_AV_ − A_AV_), reflecting the influence of the visual stimulus on the perceived location of the simultaneous sound (Fig. 1*C*, left). All group-level ve biases were significantly different from zero (*n* = 19; signed-rank test: *p* < 0.01 for all 6 ΔVA values). A generalized linear mixed model (GLMM) revealed that the ventriloquism bias varied nonlinearly with the discrepancy but did not differ between paradigms (Table 1, top section).

**Table 1.** Results from the generalized linear mixed-model analysis

Name	Estimate (β)	*t* Statistics	*p* value	CI (95%)	Model fits
Equation 1: ve ∼ β*_0_* + β*_1_* · (ΔVA)^½^ + β*_2_* · ΔVA + β*_3_* · P + β*_4_* · (ΔVA)^½^:P + β*_5_* · ΔVA:P + (1/subj)					
Intercept (ΔVA)^½^ ΔVA P (ΔVA)^½^:P ΔVA:P	−2.0029 2.0629 0.0231 0.0636 −0.1427 −0.0513	−3.2469 16.5643 0.9645 0.4070 −0.7676 −1.4370	0.0012 0.0000 0.3348 0.6840 0.4427 0.1507	−3.2120, −0.7938 1.8188, 2.3070 −0.0238, 0.0700 −0.2427, 0.3700 −0.5070, 0.2216 −0.1214, 0.0187	BIC: 109,000 AIC: 108,940 LL: −54,460
Equation 1: vae ∼ β*_0_* + β*_1_* · (ΔVA)^½^ + β*_2_* · ΔVA + β*_3_* · P + β*_4_* · (ΔVA)^½^:P + β*_5_* · ΔVA:P + (1/subj)					
Intercept (ΔVA)^½^ ΔVA P (ΔVA)^½^:P ΔVA:P	0.0017 0.8909 −0.0635 −0.0006 −0.7178 0.0733	0.0228 10.1074 −3.7469 −0.0053 −5.4568 2.8981	0.9818 0.0000 0.0002 0.9958 0.0000 0.0038	−0.1435, 0.1469 0.7181, 1.0636 −0.0967, −0.0303 −0.2172, 0.2161 −0.9757, −0.4600 0.0237, 0.1229	BIC: 98656 AIC: 98,595 LL: −49,290
Equation 2: vae ∼ β*_0_* + β*_1_* · (ΔVA)^½^ + β*_2_* · ΔVA + β*_3_* · R_AV_ + β*_4_* · *P* + β*_5_* · (ΔVA)^½^:P + β*_6_* · ΔVA*:P* + (1/subj)					
Intercept (ΔVA)^½^ ΔVA R_AV_ P (ΔVA)^½^:P ΔVA:P	0.0540 0.8369 −0.0510 0.0262 −0.0002 −0.7137 0.0746	0.7247 9.4553 −2.9898 5.8954 −0.0015 −5.4320 2.9523	0.4686 0.0000 0.0028 0.0000 0.9988 0.0000 0.0032	−0.0921, 0.2001 0.6634, 1.0104 −0.0844, −0.0176 0.0175, 0.0349 −0.2166, 0.2162 −0.9713, −0.4562 0.0251, 0.1241	BIC: 98,631 AIC: 98,562 LL: −49,272

CI, 95% confidence interval (parametric); AIC, Akaike information criterion; LL, log-likelihood. Top section reveals the linear and nonlinear dependency of the ve on multisensory discrepancy (ΔVA), which did not differ between paradigms (P). Middle section reveals the linear and nonlinear dependency of the vae on multisensory discrepancy, which both differed between paradigms. Bottom section comparing models 1 and 2 shows that some of the variance in the aftereffect is also explained by the response in the AV trial (R_AV_).

Regarding the ventriloquism aftereffect, the behavioral responses in the A trials revealed a clear bias in the direction of the ΔVA of the previous trial (Fig. 1*C*, right). All group-level vae biases were significantly different from zero (signed-rank test: *p* < 0.01 for all 6 ΔVA values). The GLMM showed that the aftereffect exhibited both a linear and a nonlinear dependency on discrepancy (Table 1, middle). Importantly, both the linear and nonlinear dependencies on ΔVA differed between paradigms (*p* < 0.01; Table 1, middle). Closer inspection of the trial-wise dynamics of these effects revealed a clear accumulation of the aftereffect over the course of the long-term paradigm but not over the short-term paradigm (Fig. 1*D*, right). The ventriloquism bias in the AV trials, in contrast, did not change over time (Fig. 1*D*, left).

### The aftereffect bias reflects the previous multisensory discrepancy

Previous studies suggested the following two potential factors driving the aftereffect: the sensory discrepancy (ΔVA) in the previous trial, or the participant's response in that trial (R_AV_) (Van der Burg et al., 2018; Park and Kayser, 2019). Indeed, in many laboratory paradigms sequential effects between the responses on different trials emerge, by which the previous response is predictive of the subsequent one (Fritsche et al., 2017; Kiyonaga et al., 2017; Talluri et al., 2018; Urai et al., 2019). We asked whether the trial-wise aftereffect biases are better accounted for by allowing a dependency on the previous response R_AV_ (Eq. 1 vs Eq. 2; Table 1, middle, bottom). The model fit improved by adding the previous response (ΔBIC = 25), suggesting that this indeed contributes to shaping the bias in the A trial in addition to the multisensory discrepancy. For the following analysis, we hence considered both ΔVA and R_AV_ as variables of interest whose cerebral representations in the AV trial could be predictive of the subsequent aftereffect.

### Multisensory neurophysiological representations driving the aftereffect

In a first analysis strand we asked whether and which EEG activations reflecting the cerebral encoding of the multisensory information (ΔVA) or the response in the AV trial are predictive of the subsequent vae bias (Fig. 2*A*). For this, we extracted EEG-derived representations of these variables using single-trial classification. We then quantified whether and which of these representations are predictive of the trial-wise vae bias. In a first analysis, we tested this within the LT and ST paradigms individually, to potentially reveal representations that are either paradigm specific or possibly exhibit common properties (time, topographies) between paradigms. In a second analysis, we directly aimed to extract EEG-derived representations that are common to both paradigms, by predicting the bias in one paradigm based on classifiers trained on the EEG activity in the other paradigm.

We applied LDA to the AV trial data to probe when the EEG activity allows the (cross-validated) classification of the two main variables of interest: ΔVA and R_AV_. Here, ΔVA served as the main variable of interest driving the aftereffect, and R_AV_ as a control. In both the LT and ST paradigms, discrimination performance became significant from ∼100 ms post-stimulus onset (Fig. 2*B*). The performances of both classifiers were significant over a long time in the LT paradigm (LDA-ΔVA: *p* = 0.0003, *t*_cluster_ = 16.71, peak = 0.87, range = 62 ms–475 ms; LDA-R_AV_: *p* = 0.0003, *t*_cluster_ = 6.70, peak = 0.73, range = 102 ms–368 ms) and the ST paradigm (LDA-ΔVA: *p* = 0.0003, *t*_cluster_ = 14.18, peak = 0.88, range = 95 ms–442 ms; LDA-R_AV_: *p* = 0.0003, *t*_cluster_ = 6.35, peak = 0.72, range = 95 ms–342 ms).

We then asked whether and when the cerebral representations of these variables are predictive of the aftereffect bias. Figure 2*C* shows the respective group-level *t* values of the regression betas from Equations 3 and 4 for the within-paradigm analysis. Figure 2*E* (left) shows the time courses of the mean rather than significance. The LDA-ΔVA predicted the subsequent trial-wise vae bias between 75 ms and 475 ms in the LT paradigm (*p* = 0.0003, *t*_cluster_ = 311.6, *t*_peak_ = 6.96, Cohen's *d* = 1.60). In the ST paradigm, the LDA-ΔVA predicted the bias between 142 ms and 202 ms (*p* = 0.001, *t*_cluster_ = 36.1, *t*_peak_ = 4.88, Cohen's *d* = 1.12; Fig. 2*C*, right), with the significant clusters overlapping between both paradigms. In contrast, the LDA-R_AV_ in the AV trial was not predictive of the bias in either paradigm (no significant clusters; maximum Cohen's *d* = 0.22, at 202 ms in the LT; *d* = 0.30 at 355 ms for the ST).

Then, in a direct cross-decoding analysis, we tested whether cerebral representations of these variables can predict the bias between paradigms (Fig. 2*D*). This revealed a significant cluster between 261 ms and 301 ms, in which the LDA-ΔVA in the AV trial of the LT paradigm predicts the bias in the A trial in the ST paradigm, and vice versa (obtained from the conjunction statistics cross-predicting in both directions; *p* = 0.0003, *t*_cluster_ = 23.8, *t*_peak_ = 3.99, Cohen's *d* = 0.91). Figure 2*E* (right) shows the time courses of the mean rather than significance.

### Distinct neurophysiological sources driving trial-wise and cumulative biases

To better understand the physiological correlates of the aftereffect biases, we extracted key time points of interest, defined as the local and global peaks in the neurobehavioral analysis (Fig. 2*C*,*D*, black dots). We then investigated the underlying neural generators by inspecting the LDA forward models and source maps. From the within-LT analysis, we derived three time points (local peaks, 115 ms and 435 ms; global peak, 241 ms). These time points were specific to the LT paradigm, as the respective EEG activity in the ST paradigm at these moments was not predictive of the aftereffect (at an uncorrected *p* < 0.01: *t* = 1.85, 1.87, 1.69; Cohen's *d*: 0.42, 0.43, 0.39). From the within-ST analysis, we derived one time point (148 ms; the LT analysis revealed a significant cluster at the same time). From the cross-paradigm analysis, we obtained one time point (global peak, 288 ms). Given that the significant clusters for the within ST and LT analysis overlapped, we asked whether the forward models of the LDA-ΔVA components were similar (at 148 ms): these were indeed highly correlated (Spearman's ρ = 0.97; bootstrap-based CI = 0.52, 0.98; *p* < 0.001), suggesting that the underlying generators are similar. We hence combined the topographies and sources across paradigms at 148 and 288 ms. The resulting forward topographies are shown in Figure 3.

Then, we asked whether the relevant neurophysiological sources were similar between time points within paradigms (LT: 115 ms vs 241 ms: ρ = −0.34, CI = −0.79, 0.29; 115 ms vs 435 ms: ρ = 0.86, CI = −0.42, 0.97; 241 ms vs 435 ms: ρ = −0.43, CI = −0.81, 0.59; ST/LT: 148 ms vs 288 ms: ρ = 0.50, CI = −0.45, 0.75). The group-level forward models were not significantly correlated between time points (all pairs *p* > 0.05, group-level bootstrap confidence intervals). This demonstrates that activity at each time point reflects distinct neurophysiological contributions to the aftereffect, suggesting a contribution from multiple and temporally dispersed processes. Furthermore, this result demonstrates that partly distinct processes contribute to the trial-wise and cumulative biases.

Finally, we inspected the underlying generators in source space. The group-level source maps revealed an involvement of medial superior parietal regions (in particular, the precuneus) at multiple time points and common to both paradigms (e.g., at 288 ms; Fig. 3*C*), in line with the hypothesis that parietal structures involved in sensory causal inference and memory mediate recalibration in general. Common to both paradigms were also sources in sensory regions (occipital and temporal cortex; at 148 ms and 288 ms), while sources specific to the LT paradigm involved precentral and frontal regions (Fig. 3*A*, Table 2).

**Table 2.** Anatomical labels of source clusters for each time point in Figure 3

115 ms LT	148 ms LT/ST	241 ms LT	288 ms ST ⇆ LT	435 ms LT
Pre-/Para-/ Post-central Precuneus Parietal inf/sup	Temporal inf Occipital inf/mid Calcarine	Frontal inf/mid/sup Supp. motor Paracentral Parietal Sup	Temporal inf/mid Cuneus Precuneus Angular Calcarine Occipital inf/mid/sup	Frontal sup Supp. motor pre-/para-/ Post-central Parietal inf/sup

Anatomical labels are based on the AAL atlas (Tzourio-Mazoyer et al., 2002). See Materials and Methods for the extraction of these regions. inf: inferior, sup: superior, mid: middle, Supp: Supplementary.

### Trial-wise and cumulative effects manifest differentially in auditory trials

In a second analysis strand, we asked whether and which EEG activations reflecting the encoding of the task-relevant acoustic information in the A trial are biased by the previously experienced multisensory information (ΔVA) or the previous response (R_AV_; Fig. 4*A*). This analysis follows the logic set out in previous work, where it has been speculated that trial-wise and cumulative aftereffects emerge with different latencies in neurophysiological activity during the A trial (Bruns et al., 2011; Zierul et al., 2017; Park and Kayser, 2019).

Classification performance of the current sound location in the A trial was significant from ∼100 ms onward and over an extended time window in both paradigms (LT: *p* = 0.0003, *t*_cluster_ = 3.6, peak = 0.63, range = 61 ms–281 ms; ST: *p* = 0.0001, *t*_cluster_ = 4.5, peak = 0.69, range = 101 ms–341 ms; Fig. 4*B*, left). Given that task and stimuli were identical in both paradigms we expected that the underlying cerebral representations of sound position would be the same and hence allow for cross-classification. Indeed, cross-classification was significant in the same time window (*p* = 0.0002, *t*_cluster_ = 4.64, peak = 0.66, range = 48 ms–341 ms; Fig. 4*B*, right) and the classifier topographies (Fig. 5) at the time of local peaks in the cross-decoding performance were significantly correlated between paradigms (at 141 ms: Spearman's ρ = 0.89, bootstrap-based CI = −0.202, 0.98, *p* = 0.015; at 268 ms: ρ = 0.82, CI = −0.05, 0.96, *p* = 0.009).

We then asked whether and when these representations of sound position are biased by the multisensory discrepancy experienced in the previous AV trial, or the motor response in that trial (Eqs. 5, 6). This revealed a significant influence of ΔVA on sound encoding in the A trial at different time points in each paradigm (Fig. 4*C*). For the LT paradigm, this effect emerged early in the trial (cluster 115 ms−148 ms, *p* = 0.001, *t*_cluster_ = 22.1, *t*_peak_ = 4.35, Cohen's *d* = 1.00), while for the ST paradigm the effect emerged considerably later (228 ms−248 ms, *p* = 0.012, *t*_cluster_ = 13.9, *t*_peak_ = 4.10, Cohen's *d* = 0.94), suggesting that the neurophysiological processes affected by the multisensory discrepancy differ between paradigms. Indeed, the attempt to directly predict this influence of multisensory discrepancy on sound encoding using cross-classification did not yield any significant effects (no significant clusters; maximum Cohen's *d* = 0.41; Fig. 4*D*). In addition, the topographies of the classifiers at the respective two time points of peak effects were distinct between paradigms (comparing the LDA forward models for LT at 135 ms and ST at 235 ms: ρ = 0.15, CI = −0.77, 0.70, *p* = 0.487; Fig. 5), and the effect size for ΔVA to bias sound representations in the respective other paradigm at the time of each paradigm-specific local peak was considerably smaller (Cohen's *d* at the peak time of significance in the LT paradigm, 135 ms, computed in the ST data were *d* = 0.23; vice versa at the peak time of significance in the ST paradigm, 235 ms, computed in the ST data *d* = 0.67). Finally, when we applied same analysis using the previous motor response as predictor of sound representations, we found no significant effects (no significant clusters; maximum Cohen's *d* = 0.51 for LT; maximum Cohen's *d* = 0.42 for ST). This shows that the impact of the previously experienced multisensory information during the auditory trial emerges in distinct spatial sources and with different timing for trial-wise and cumulative multisensory exposure.

Finally, we also inspected the underlying generators in source space. Given that the forward models of the discriminant components for sound location were highly correlated between paradigms, we averaged the resulting sources across paradigms (Fig. 5). The group-level sources were broadly distributed but encompassed parietal and temporal regions at the time points relevant for each paradigm (e.g., at 135 ms for LT, 235 ms for ST; Fig. 4*C*). For the ST paradigm the sources also reveal a more prominent involvement of frontal regions (Table 3).

**Table 3.** Anatomical labels of source clusters for each time point in Figure 5

135 ms LT		235 ms ST	
Temporal inf/mid/sup Occipital inf/mid/sup Cuneus Precuneus Parietal inf/sup	Calcarine Angular Lingual	Temporal Mid/Sup Pre-/post-central Frontal inf/mid Parietal inf Supramarginal	Rolandic Operculum Angular

Anatomical labels are based on the AAL atlas (Tzourio-Mazoyer et al., 2002). See Materials and Methods for the extraction of these regions inf: inferior, mid: middle, sup: superior.

### Eye movements do not confound these results

To ensure that potential eye movements do not confound our conclusions, we analyzed both eye movements themselves and their predictive power for the single-trial biases (Werner-Reiss et al., 2003; Kopco et al., 2009). First, only a few A trials (mean ± SEM; 2.3% ± 0.5% across participants and trials) contained saccadic eye movements during stimulus presentation (>1°; between −50 ms and 100 ms of stimulus onset), showing that participants maintained fixation well. Second, we computed the percentage of saccades in AV trials between stimulus onset and 400 ms that pointed in the same direction as ΔVA, and hence would directly confound with the direction of ΔVA as a predictor. Overall, the direction of saccades was very balanced: with only 51.2% ± 3.1% (mean ± SEM; LT, 52.0% ± 3.1%; ST, 50.3% ± 3.2%) pointing in the same direction as ΔVA. Finally, to rule out the possibility that eye movements contribute by inducing specific artifacts to the EEG analysis, we repeated the above analyses using the EOG signals instead of the EEG activity. These analyses did not provide any significant relation between any putative information about ΔVA in the EOG signals and the vae bias (in analogy to Fig. 2) or between information about the sound location in the A trial and the previously experienced discrepancy (based on the same statistical criteria as for the EEG data, no clusters emerged at *p* < 0.05).

## Discussion

We often encounter seemingly discrepant multisensory information, such as when watching a movie on a screen while hearing sounds through earphones. Our brain reconciles such discrepant information by adapting to the sensory disparity over multiple timescales. While previous work suggested that the ventriloquism aftereffects from cumulative and trial-wise exposure arise from distinct mechanisms, the evidence has been indirect and mostly from behavioral studies (Frissen et al., 2012; Bruns and Röder, 2015; Van der Burg et al., 2015; Bosen et al., 2018). We here directly tested within the same participants whether both aftereffects are shaped (while presented with discrepant multisensory evidence, AV trial) and implemented (while presented with a subsequent unisensory test stimulus, A trial) by the same neurophysiological processes. Our results show that while presented with the multisensory evidence, both aftereffects are shaped by common neurophysiological correlates arising from sensory and parietal regions, while prolonged exposure additionally recruits frontal regions. During the subsequent unisensory test trial, however, the trial-wise and cumulative aftereffects are mediated by distinct neurophysiological processes reflecting the biased encoding of the current sound location at shorter (cumulative bias) and longer (trial-wise bias) latencies.

### The neural underpinnings of the spatial ventriloquism aftereffect

Sensory recalibration as in the ventriloquism aftereffect is robustly seen across variations of the paradigm and after exposure over multiple timescales (Recanzone, 1998; Lewald, 2002; Wozny and Shams, 2011; Bruns and Röder, 2015, 2019; Mendonça et al., 2015). Although it is conceptually difficult to strictly separate trial-wise and cumulative effects, in particular as the latter always encompasses some of the former, behavioral studies suggested that trial-wise and cumulative biases obtained after prolonged exposure times arise from distinct mechanisms, in particular as the trial-wise bias generalizes across stimulus features (e.g., sound frequencies) while the cumulative effect seems more specific (Recanzone, 1998; Lewald, 2002; Bruns and Röder, 2015, 2019). Yet, the degree of stimulus specificity remains debated (Frissen et al., 2003, 2005), and, by nature, the behavioral studies remain inconclusive about the precise neural underpinnings.

Previous EEG studies found that prolonged exposure to audiovisual discrepancies alters evoked responses ∼100 ms following stimulus onset and suggested a neural correlate near auditory cortex (Bruns et al., 2011), which is in line with single neuron data (Recanzone, 1998; Recanzone et al., 2000). Previous work also proposed that the cumulative aftereffect may be mediated by a larger temporoparietal network involved in multisensory integration, although direct evidence so far has been scarce (Zierul et al., 2017). The origin of the trial-wise ventriloquism aftereffect, in contrast, has been attributed to medial parietal regions involved in spatial working memory and sound localization (Park and Kayser, 2019), raising the question as to whether the trial-wise and cumulative aftereffects indeed are mediated by shared or distinct processes.

To reconcile the previous work, we combined two analytical approaches to test for neurophysiological processes underlying the aftereffect biases. In one approach, we followed previous studies, which either explicitly focused on auditory cortices or relied on neural signatures of sound encoding, to probe for an influence of the discrepant audiovisual information on the encoding of subsequent unisensory information (Recanzone, 1998; Recanzone et al., 2000; Zierul et al., 2017). By design, this approach likely reveals neural processes involved in auditory encoding, while those concerned with other computations such as multisensory fusion or sensory causal inference are less likely to emerge. Hence, we also considered a second approach that focused on neural processes reflecting the encoding of the multisensory spatial discrepancy, which is the key driver of the aftereffect (Wozny and Shams, 2011; Park and Kayser, 2019).

Combining these two approaches allowed us to directly confirm the notion of a partly shared neural substrate shaping the aftereffect, whereby sensory and parietal regions encode and maintain information about the multisensory environment to guide adaptive behavior. However, during the unisensory test trial, the trial-wise and cumulative aftereffect arise from neural signatures of biased sound representations at different latencies and from different sources, in line with both biases being implemented by distinct circuits. Confirming previous EEG results, the aftereffect emerged at ∼100 ms from sound onset following cumulative exposure, possibly reflecting changes in auditory cortical sound encoding because of processes reflecting adaptation or perceptual learning on longer timescales and possibly mediated by top-down guidance (Bruns et al., 2011; Bruns and Röder, 2015; Zierul et al., 2017). In contrast, the aftereffect for trial-wise exposure emerged at much longer latencies (>200 ms). Several forms of sequential patterns in behavior have been attributed to longer-latency and cognitive processes, and our previous MEG study implied parietal activity beyond ∼130 ms from stimulus onset in the trial-wise ventriloquism effect (Park and Kayser, 2019). The collective evidence from the present and previous work hence suggests that the trial-wise bias arises from higher-level regions beyond immediate sensory encoding. Future work could seek to confirm these common and distinct neural processes mediating the two timescales of the ventriloquism aftereffect using brain stimulation geared to selectively interfere with the putative shared or common process and establishing a causal role of these.

### A potential role of motor history in the aftereffects

Previous work suggests that adaptive recalibration may not be a purely sensory phenomenon but is also driven by participants' previous motor reports (Van der Burg et al., 2018; Park et al., 2020). That is, both the audiovisual stimulus and participant's response during the AV trial could contribute to the aftereffect bias in the A trial. While several studies reported a stronger sensory than motor influence on the ventriloquism aftereffect (Van der Burg et al., 2018; Park and Kayser, 2019; Park et al., 2020), here the influence of the previous motor response on the behavioral bias was significant. Hence, we also probed whether neurophysiological signatures of the previous response (R_AV_) were predictive of the aftereffect. However, we found no such effects. This corroborates the predominant sensory nature of spatial ventriloquism and rules out motor-related confounds as mediators of the reported neurophysiological underpinnings.

### Multiple timescales of the ventriloquism aftereffect

Our results consolidate previous work by showing that the trial-wise and cumulative aftereffects are shaped by shared neural processes reflecting the encoding of discrepant multisensory information in sensory and parietal regions. Previously, using MEG-based source imaging, we found that the trial-wise effect is mediated by medial parietal regions (Park and Kayser, 2019) involved in spatial working memory and sound localization, such as the precuneus (Lewald et al., 2008; Tao et al., 2015)(Martinkauppi et al., 2000). While the precise sources underlying the present EEG data have to be interpreted with care, they are compatible with the same parietal regions mediating the ventriloquism aftereffect over multiple timescales, highlighting that structures involved in working or procedural memory play a central role for this form of adaptive behavior (Ester et al., 2015; Müller et al., 2018; Schott et al., 2019).

The cumulative bias was also shaped by a more extensive network involving precentral and frontal regions. Previous work implied inferior frontal regions in multisensory causal inference (Rohe and Noppeney, 2015; Cao et al., 2019). During prolonged exposure the multisensory discrepancy follows a regular pattern, while it is seemingly random in the trial-wise paradigm. A systematic pattern allows the formation of predictions about upcoming stimuli and may drive the formation of working memory about sensory causal relations (Curtis, 2006; Noppeney et al., 2010; Collins and Frank, 2013; Nee and D'Esposito, 2016). One possibility is that parietal regions guide the aftereffect based on the more immediate stimulus history, while frontal processes exploit the regularity over longer timescales. Such a divided role fits with the notion that parietal regions contribute to the immediate fusion of multisensory information within a trial, while frontal regions help differentiating whether two stimuli arise from a common source (Rohe and Noppeney, 2015; Cao et al., 2019; Rohe et al., 2019), a process known to benefit from knowledge about stimulus history (Beierholm et al., 2019). Future work should investigate whether the same or distinct frontal regions contribute to causal inference within a trial and the fostering of recalibration based on the cumulative stimulus history.
